# Laser-Prepared ZnO-Ag Nanoparticles with High Light-Enhanced Antibacterial Activity

**DOI:** 10.3390/ma18133088

**Published:** 2025-06-29

**Authors:** Anastasia V. Volokitina, Elena D. Fakhrutdinova, Daria A. Goncharova, Sergei A. Kulinich, Valery A. Svetlichnyi

**Affiliations:** 1 Laboratory of Advanced Materials and Technology, Tomsk State University, 36 Lenina Ave., Tomsk 634050, Russia; 2 Research Institute of Science and Technology, Tokai University, Hiratsuka 259-1292, Kanagawa, Japan

**Keywords:** ZnO-Ag nanoparticles, defects, photocatalytic antibacterial activity, light bacterial inactivation

## Abstract

Recently, the urgency of combating antibiotic-resistant bacteria, viruses, and other pathogens has dramatically increased. With the development of nanotechnology, significant hopes are placed on nanoparticles with antimicrobial properties. The efficiency of such materials can be significantly enhanced through light-activated processes. In this study, we prepared composite ZnO-Ag nanoparticles and tested their ability to inhibit *Staphylococcus aureus* bacteria. The composite ZnO-Ag nanoparticles were fabricated using pulsed laser ablation of Zn and Ag targets in water using a nanosecond pulsed laser. During antibacterial tests, light-enhanced activation of the nanoparticles was achieved using low-power near UV (375 nm) and blue visible (410 nm) LED irradiation. For comparison, similar laser-fabricated ZnO nanoparticles were also tested. The combined use of nanoparticles and LED irradiation significantly increased the generation of reactive oxygen species. As a result, low nanoparticle concentrations (0.05 g/L) and low-power LED irradiation (0.17–0.22 W) significantly reduced the concentration of *Staphylococcus aureus* bacteria, including experiments with visible light irradiation. Compared to their ZnO counterparts, the use of ZnO-Ag composite particles led to an additional increase in antimicrobial activity.

## 1. Introduction

The rapid development of industrial production and agriculture, along with population growth and increased consumption, has significantly increased water pollution [1,2,3,4,5]. This pollution includes not only various organic compounds [1,2,3] but also potentially dangerous microorganisms [4,5]. Additionally, the expansion of the pharmaceutical industry, particularly the rise in antibiotic production and their uncontrolled use, has led to the emergence of antibiotic-resistant bacteria [6,7], posing a potential threat to human life. Technologies for water purification and disinfection using optical radiation, including photocatalytic methods, have been utilized for many years, and these days, photocatalytic processes are recognized as cost-effective and accessible water purification technologies [8]. Photocatalytic oxidation and decomposition of organic pollutants using semiconductors as catalysts have proven to be effective [9,10], while research on the photocatalytic inactivation of bacteria is rapidly advancing [11,12,13].

The generation of reactive oxygen species (ROS) by semiconductor particles under light exposure is known to be an effective method for eliminating pathogenic bacteria [14]. Nanoparticles (NPs) of various semiconductor oxides [15,16] and more complex structures based on these oxides [17,18,19,20] are employed as photocatalysts to inactivate bacteria. Among them, nanosized TiO_2_ and ZnO are the most frequently used materials [21,22,23,24], which is explained by their relative availability, the development of various synthesis methods, non-toxicity to human cells [25], and their widespread application in biomedical engineering (bioimaging, wound healing, implant coating, tissue engineering, and anti-cancer drug discovery) [26]. ZnO NPs exhibit significant antibacterial activity against a wide range of pathogenic bacterial species [27,28], and their photocatalytic antibacterial activity is directly dependent on their NP size [29].

Since ZnO is a wide-gap semiconductor (*E*_g_~3.3 eV), UV irradiation is required to generate electron-hole pairs, which subsequently produce ROS. Several approaches can help expand the spectral absorption range of semiconductors, one of which involves modifying them with noble metals (Au, Ag) that exhibit surface plasmon resonance (SPR) in the visible spectrum [30,31,32]. Adding a noble metal with a high electron work function to a semiconductor NP also leads to the formation of a Schottky barrier, enhancing the separation of photogenerated charge carriers [33,34]. When such composite NPs are excited within the SPR absorption region, the rate of photoinduced charge carrier formation significantly increases [35,36], thereby boosting the concentration of ROS generated during irradiation [37].

Another effective approach to increasing antibacterial activity during photoactivation is the formation of defects in the ZnO structure, which enables absorption in the visible spectrum [38,39]. Recent studies have demonstrated that creating defective ZnO NPs enhances the generation of reactive oxygen species [40,41].

Pulsed laser ablation (PLA) is considered one of the most promising methods for obtaining highly efficient functional nanomaterials for catalysis and biomedicine [42,43]. Its relative simplicity, environmental friendliness, and high variability in the solvents used make this method unique for synthesizing both simple semiconductor systems and complex composite structures [42]. Additionally, during PLA synthesis, rapid changes in local heating and cooling in the liquid cause the formation of various types of defects in semiconductor structures, including sensitization to low-energy photons due to such defects [44,45,46]. The preparation of ZnO NPs and nanostructures for photocatalysis and biomedicine is widely studied and remains in high demand [26,47,48,49]. We have previously prepared defective ZnO NPs under various conditions [46], including a composite modified with Ag NPs [50], for use as photocatalysts in the decomposition of various organic compounds (dyes, phenol, antibiotics) to purify aqueous media and as antibacterial agents. The nature of defects and methods to control their concentration have also been studied in detail [45,46].

Although the antibacterial properties of ZnO-Ag composites and their efficacy against *Staphylococcus aureus* under irradiation have been the subject of considerable research, many of the previous studies typically utilized broad-spectrum light sources, high-power UV radiation, or high concentrations of NPs [51,52,53,54,55,56]. In contrast, the present study is characterized by a comprehensive approach based on the targeted preparation of defective ZnO to enhance its photocatalytic activity, the use of minimal silver loading (1 wt.%) to optimize plasmonic effects, demonstration of efficiency at extremely low NP concentrations (0.05 g/L), and activation by low-power LEDs at specific wavelengths (375 nm and 410 nm). Developing on our previous studies devoted to the characterization and application of PLA-derived defective ZnO [45,46] and ZnO-Ag [50] systems for the degradation of organic pollutants, the present work focuses on their light-enhanced antibacterial potential under conditions as close as possible to practical applications and characterized by “soft” irradiation. Accordingly, the aim of the present research was to enhance the antibacterial activity of the final composite ZnO-Ag material by leveraging the photocatalytic properties of semiconductor NPs under conditions of mild, safe photoirradiation, and potentially reduce the concentration of particles used.

## 2. Materials and Methods

### 2.1. PLA Synthesis of ZnO and ZnO-1Ag NPs

Antibacterial NPs were synthesized via pulsed laser ablation (PLA) of high-purity (99.9%) Zn and Ag metal targets in distilled water. The process used an Nd:YAG laser (LS2131M-20, LOTIS TII, Minsk, Belarus) with the following radiation parameters: wavelength (λ) of 1064 nm, pulse duration of 7 ns, pulse energy of 150 mJ, and repetition frequency of 20 Hz. The concentration of NPs in produced colloids was determined based on the mass loss of the target (evaluated via comparing the mass before and after ablation).

A zinc oxide sample was synthesized via PLA of a high-purity Zn target in 80 mL of distilled water for 30 min. Its average NP concentration was approximately 0.3 mg/L. The resulting colloidal solution was air-dried at ~60 °C, and the obtained powder was calcined at 400 °C for 4 h in a muffle furnace. During annealing, zinc hydroxycarbonate Zn_2_(CO_3_)_2_(OH)_6_, formed as an inactive impurity phase (1–3%) during synthesis and drying, decomposed. More detailed XRD patterns and TG-DSC analyses were presented elsewhere [50].

To prepare the ZnO-Ag composite material, individual colloidal solutions of ZnO and Ag NPs were first prepared via PLA of Ag and Zn targets, after which they were mixed at a mass ratio of Ag to ZnO of 1:99. The resulting colloid was processed in an ultrasonic bath for 15 min, air-dried at 60 °C, and subsequently calcined at 400 °C. The final NP samples were labeled as ZnO and ZnO-1Ag.

### 2.2. Characterization Methods

The crystal structure of NPs was determined by X-ray diffraction (XRD) on an XRD-7000 diffractometer (Shimadzu, Kyoto, Japan) with monochromatic CuK_α_ radiation (1.54 Å) in the 2*θ* range of 10–80° and with a scanning rate of 0.02 °/s. Data were obtained using the Bragg-Brentano geometry. Crystalline Si (*a* = 5.4309 Å, *λ* = 1.540562 Å) was used as an external standard to calibrate the diffractometer. The phase composition was analyzed using the PDF-4 database. To refine the crystal lattice parameters and determine coherent scattering regions (CSRs) for crystalline phases, the full-profile analysis program POWDER CELL 2.4 was used.

The Ag content in the composite ZnO-1Ag NPs was confirmed using an XRF-1800 X-ray spectrometer (Shimadzu, Kyoto, Japan) with preliminary calibration using specially prepared mixtures of ZnO and Ag.

The size and shape of individual particles were assessed by transmission electron microscopy (TEM) on a JEM-2100 (JEOL Ltd., Tokyo, Japan). For studies, the samples were dispersed in ethanol and placed on copper grids coated with carbon film. Microscopic studies and analysis of the elemental composition of the powder surface were carried out on the Vega 3 SBH scanning electron microscope (Tescan, Brno, Czech Republic) with a thermionic tungsten cathode and the AztecLive Lite Xplore 30 energy-dispersive microanalysis system (Oxford Instruments, Abingdon, UK).

The specific surface area of the NPs was determined using the gas adsorption analyzer of specific surface area and porosity, TriStar II 3020 (Micromeritics, Norcross, GA, USA), by low-temperature nitrogen sorption. Before the analysis, the samples were degassed in a vacuum (10^−2^ Torr) at 150 °C for 2 h.

The optical properties of the materials were analyzed using diffuse reflectance spectroscopy (DRS) on a Cary 100 spectrophotometer equipped with the DRA-CA-30I Labsphere module, covering a wavelength range of 230–800 nm, while MgO was used as the measurement standard. To calculate the optical band gap, the spectra were rearranged into coordinates (*F*(*R*)*hv*)^2^-*E*(eV) using the Tauc method; the values were determined by extrapolating a straight line to the *X*-axis. Photoluminescence (PL) spectra were recorded on a Fluorolog 3–22 spectrofluorimeter (Horiba, Jobin Yvon, Edison, NJ, USA) in the wavelength range of 280–750 nm.

### 2.3. Antibacterial Activity of NPs and of Irradiation

*Staphylococcus aureus* (*S. aureus* ATCC 6538) was used as a test microorganism. The work was carried out with a daily inoculum of *S. aureus*, which was prepared in a sterile nutrient medium (SMM) by incubation in a thermostat at a temperature of 37 °C.

Antibacterial activity tests were carried out in 250 mL glass flasks containing 50 mL of sterile sodium phosphate buffer saline (PBS) or pancreatic hydrolyzate of fish meal (PHFM) nutrient medium (agar). The concentration of *S. aureus* bacteria when studying bacterial growth in a nutrient medium was 10^4^ CFU/mL and 10^6^ CFU/mL when studying bacterial survival in PBS. The concentration of ZnO and ZnO-1Ag NPs was varied from 0 to 1 g/L, depending on the purpose of the study. Sterilization of the synthesized NP samples was carried out by irradiation with a quartz lamp for 15 min.

**Figure 1:**
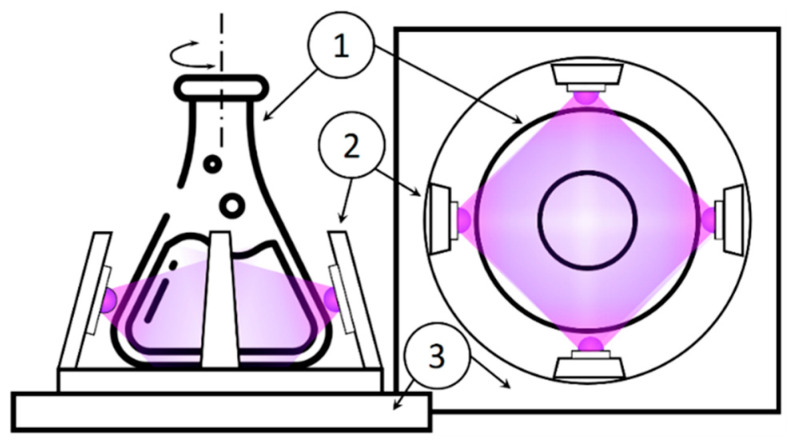
A schematic of unit used to irradiate the medium with bacteria: 1: 250 mL flask with 50 mL solution (PBS/culture medium, *S. aureus*, NPs), 2: LED, 3: shaker.

Low-intensity light-emitting diodes (LEDs), i.e., near UV with a wavelength of 375 nm (LED375) and blue Vis 410 nm (LED410), were chosen as irradiation sources. Figure 1 shows a diagram of the irradiation installation, with four LEDs used for irradiation. The radiation power was determined using a calibrated semiconductor detector PD300UV (Ophir, Tel Aviv, Israel). The medium was mixed in a laboratory shaker at 110–120 rpm and thermostatically controlled at 37 °C.

To minimize error and maintain similar experimental conditions, four parallel measurements were simultaneously carried out for samples prepared from the same bacterial culture of *S. aureus*: in medium without NPs and without irradiation, in medium with NPs without irradiation, in medium without NPs with irradiation, and in medium with NPs and with radiation.

The concentration of bacteria was determined by the method of bacteriological plate culture, with a decrease in the concentration in physiological solution on agar media in Petri dishes. After sowing in a thermostat at a temperature of 37 °C, colonies grew within 24 h and were counted manually. The concentration (CFU) after dilution and sowing was determined by the formula:

$$CFU=\frac{a\cdot 10^{n}}{V},$$

where *α* is the number of colonies in a Petri dish, *n* is the order of dilution, and *V* is the volume of inoculum in mL (in our experiments being 0.1 mL).

To determine the percentage of bacterial survival, the percentage of the number of bacteria after exposure to the initial average concentration of bacteria was used.

$$\%survivors=\frac{Number\;of\;surviving\;colonies\;CFU}{Initial\;number\;of\;colonies\;CFU}\times 100\%$$

## 3. Results and Discussion

### 3.1. Materials Characterization

The phase composition of the powders was analyzed using XRD. Table 1 presents the results, while the corresponding XRD patterns are provided in the Supplementary Materials (Figure S1). The ZnO sample was found to be represented by a single-phase, hexagonal zinc oxide with a wurtzite structure (PDF Card # 04-008-8198). In the ZnO-1Ag composite sample, in addition to wurtzite-phase reflections, additional reflections appeared around 38° (2*θ*), corresponding to the cubic phase of metallic silver (PDF Card # 04-003-1472). The silver content in the sample, further confirmed by X-ray fluorescence, was close to the stated 1 wt.%. Table 1 also shows the crystallite sizes calculated from the coherent scattering region (CSR) of the main phase of ZnO (wurtzite) and specific surface area values. For the ZnO sample, the CSR was 43 nm, and its specific surface area was 21 m^2^/g. The addition of silver was observed to prevent the coarsening of NPs during annealing, and the CSR value for the composite sample ZnO-1Ag was 37 nm, while its specific surface area was 26 m^2^/g. Table 1 presents the wurtzite hexagonal lattice constants for both samples. The modification of ZnO with Ag did not result in significant changes in these values, indicating that silver was not incorporated into the semiconductor structure but was instead distributed over the surface.

**Figure 2:**
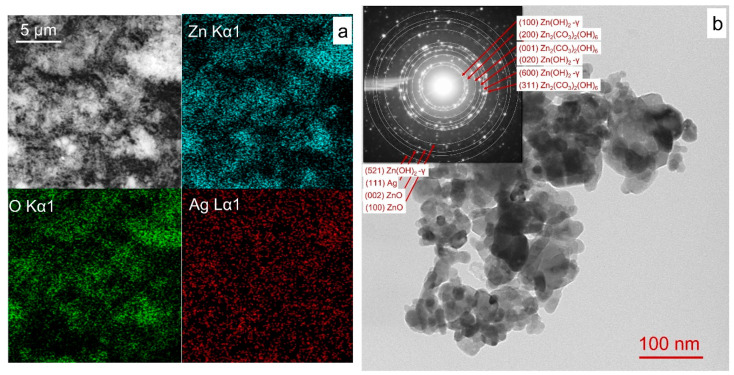
SEM images with EDX distribution of elements (**a**), TEM and SAED (**b**) for sample ZnO-1Ag.

The surface morphology and elemental distribution of powder ZnO-1Ag were analyzed using scanning electron microscopy (SEM) combined with energy-dispersive X-ray spectroscopy (EDX). The results indicated that silver was uniformly distributed throughout the sample (Figure 2a). TEM analysis indicated that the sizes and shapes of NPs in samples ZnO and ZnO-1Ag were comparable. Figure 2b presents the data for the ZnO-1Ag sample, where agglomerates consisting of particles with an average size of 30–35 nm are seen, which is consistent with CSR data. Individual larger particles up to 80 nm in size were also found. TEM analysis of the colloidal Ag solution after PLA was previously studied and presented in work [57], where Ag NPs with average particle sizes of 20–25 nm were reported. The results of selected area electron diffraction (SAED) confirmed the presence of wurtzite ZnO (reflections of the (100) and (002) planes) and the cubic Ag (metallic) phase (reflection of the (111) plane). Reflections of the crystallographic planes (100), (020), and (600), (521) of the phase of unstable γ-Zn(OH)_2_ and (200), (001), (310) of the zinc hydroxycarbonate Zn_2_(CO_3_)_2_(OH)_6_, which do not appear in the XRD data. Local formation of small amounts of Zn_2_(CO_3_)_2_(OH)_6_ and γ-Zn(OH)_2_ phases, which are thermally unstable and decompose during annealing, was most likely due to the interaction of zinc oxide with carbon dioxide and air water vapor during sample storage.

**Figure 3:**
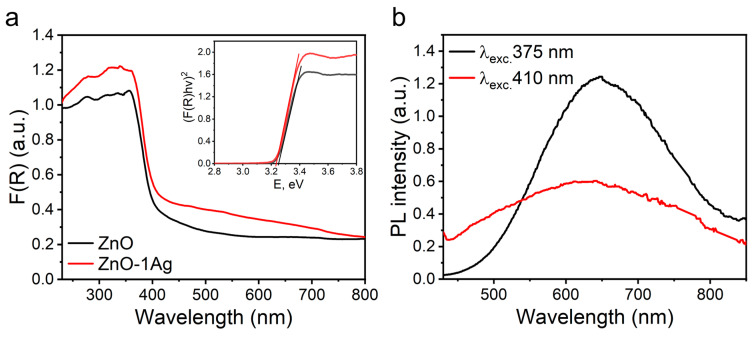
Absorption spectra for obtained powders and calculation of their band gap (inset) (**a**), photoluminescence spectra for ZnO NPs under different excitation (**b**).

Figure 3a presents the UV-Vis spectra derived from diffuse reflectance spectra using the Kubelka-Munk function. In the ZnO sample, the absorption band edge appears blurred, most likely due to the presence of defects of various types [45,46]. When silver was added, additional absorption appeared in the spectra in the region of 410–480 nm, which is associated with the surface plasmon resonance of Ag [58]. It is known that the plasmonic peak is sensitive to the size and shape of metal NPs as well as to the refractive index of the medium in which they are dispersed [59]. Small spherical Ag NPs were reported to exhibit a narrow SPR band in the region of 390–430 nm [60], while in the ZnO-1Ag nanocomposite, this band is greatly broadened and shifted towards higher wavelengths, which can be due to the strong interfacial electronic interaction between Ag and ZnO NPs and uniform distribution of fine Ag particles over the ZnO surface [61,62]. The edge of the exciton absorption band of the samples, related to ZnO, is located in the region of 380 nm and does not shift when Ag is added. The band gap estimated by the Tauc method was ~3.25 eV (see Table 1).

The nature of the defect states of ZnO NPs was investigated by the photoluminescence (PL) method. Previously, we studied the nature of defect states in ZnO obtained by PLA depending on the environment in which their synthesis was carried out (H_2_O, air) as well as subsequent heat treatment [45,46]. The present study focuses on the excitation of defects using wavelengths (375 and 410 nm) that are later employed for photocatalytic inactivation of antibacterial activity (Figure 3b). Under such excitation, a broad PL band appears in the 500–800 nm region, corresponding to defect states of various types. Sample ZnO produced by PLA features high defectiveness. Such a wide spectrum is formed by both donors, namely, interstitial zinc in the ground and positively ionized states and vacancies of various types, and acceptors, i.e., negatively charged zinc vacancies and interstitial oxygen O_i_, including excess defects [45,63]. In this case, since the spectrum is redshifted (a broad maximum in the region of 600–700 nm), they are predominantly associated with the transitions between the conduction band and interstitial ions of excess oxygen, as well as interstitial zinc ions in various states [45,64,65]. The bands in the region 480–600 nm are associated with the transitions between oxygen vacancies of various types and the valence band and transitions from the conduction band to defect levels associated with zinc vacancies [45,65,66]. In both cases of excitation, luminescence bands (both near UV and blue Vis) indicate the presence of a large number of defects and the manifestation of various channels for charge carrier transitions.

### 3.2. Bacterial Inactivation

#### 3.2.1. Evolution of Bacteria in PHFM

**Figure 4:**
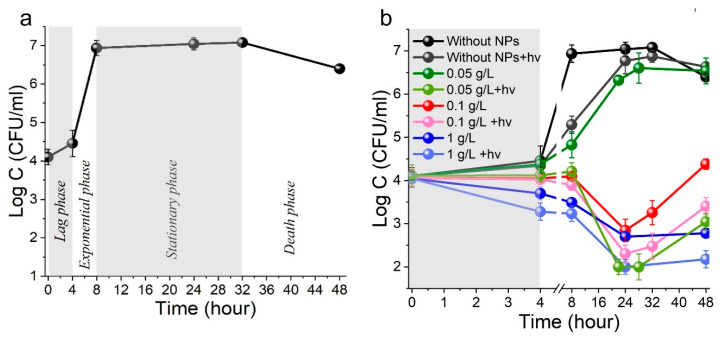
Evolution of bacteria in PHFM (**a**). Effects of ZnO NPs and irradiation on the growth of *S. aureus* bacteria in culture medium; optical power irradiation of LED 375 is 0.38 W (**b**).

In the bacterial inactivation study, the evolution of the *Staphylococcus aureus* population was initially tested in a closed nutrient medium without nanoparticles (NPs) and without irradiation (Figure 4a). Based on previous reports [67,68], the bacteriological growth curve can be divided into four main phases:

(i) The lag phase [68,69], in which bacteria increase in size but do not actively divide. Under our experimental conditions, the lag phase of bacterial evolution was about 4 h.
(ii) The exponential phase, in which rapid population growth occurs (the number of bacteria doubles at regular intervals). The exponential phase of the evolution of *S. aureus* bacteria in our case lasted from 4 to 8 h.
(iii) The stationary phase, where the number of new cells in each time interval is equal to the number of cells that die in the same time interval [68]. This was observed over the next 24 h with a bacterial concentration of 10^7^ CFU/mL.
(iv) The death phase occurs when nutrients in the growth medium are depleted, leading to a decline in the bacterial population. In our experiment, this phase was observed beginning on the second day of *Staphylococcus aureus* evolution.

#### 3.2.2. Influence of ZnO NP Concentration and LED 375 Irradiation on Bacterial Inactivation

Experiments were conducted to evaluate the influence of NP concentration, near-UV irradiation, and their combined effects on *Staphylococcus aureus* survival in a nutrient medium. NP loading concentrations were set at 1 g/L, 0.1 g/L, and 0.05 g/L, with an initial bacterial concentration of approximately 10^4^ CFU/mL. Bacterial colonies were exposed to radiation for the first 4 h, corresponding to the lag phase (Figure 4b). At an NP loading of 0.05 g/L, the bacterial growth rate was reduced. Increasing the loading to 0.1 g/L induced a bacteriostatic effect, while a further increase to 1 g/L resulted in an antibacterial effect, leading to bacterial death.

Irradiation with soft near-UV radiation of 375 nm without ZnO NPs was found to slightly reduce the growth of *S. aureus* colonies, and when combined with NPs and LED375 radiation, bacterial death occurred even at the ZnO concentration of 0.05 g/L. With an increase in the concentration of NPs to up to 1 g/L, the number of bacteria in the nutrient medium dropped by an order of magnitude. Summarizing the observed results, one can conclude that the bacteriostatic effect was observed at a concentration of 0.1 g/L NPs without LED375 radiation, and the bactericidal effect at a concentration of 1 g/L NPs without LED375 radiation or 0.05 g/L NPs with LED375 radiation.

Then, the survival of bacteria in the presence of NPs was monitored after LED375 irradiation for 2 days. In the presence of ZnO NPs with a low loading of 0.05 mg/L, as well as with the LED375 irradiation without NPs, we observed a slowdown in bacterial evolution and a decrease in active growth during the exponential phase in the subsequent 24 h compared to the initial experiment without exposure. In the next 20 h, we observed the stationary phase, where the concentration of *S. aureus* reached ~10^6^ CFU/mL. With a higher loading of NPs (0.1 and 1 g/L) as well as with simultaneous exposure to any loading (0.05, 0.1, and 1 g/L) of NPs and LED375 irradiation in the 18–20 h following the lag phase, the intense bacterial death occurred, the concentration of *S. aureus* colonies decreased to ~10^2^–10^3^ CFU/mL. Then, for 2–4 h, for most experiments, there was a stationary period, which can be attributed to the appearance of a new lag phase, after which an exponential phase of bacterial growth was observed. A high concentration of NPs (1 g/L), both in the case of LED375 exposure and without, led to a bacteriostatic effect; in the next 20 h, no bacterial growth was observed in the nutrient medium.

Since the NPs at a loading of 0.1 and 1 g/L, even without LED375 irradiation, featured a bacteriostatic and antibacterial effect, for further studies of the combined effect of ZnO and irradiation on the inactivation of bacteria, an NP loading of 0.05 g/L was chosen, and this concentration was used in further experiments. Also, in the subsequent experiments, to study the irradiation effect on antibacterial activity, the systems in physiological solution without a nutrient medium were used. In such a system, the concentration of bacteria did not change within 4 h, and the system remained stable (Figure S2).

#### 3.2.3. Effects of Wavelength and Power of LED Irradiation

**Figure 5:**
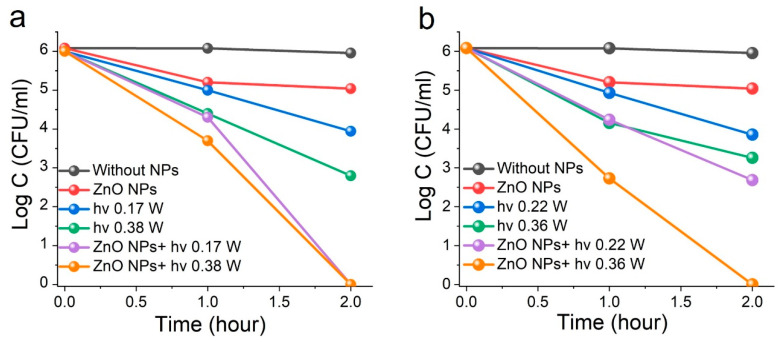
Inactivation of *S. aureus* bacteria upon exposure to 0.05 g/L ZnO NPs and irradiation of different wavelengths and power: (**a**) LED 375, (**b**) LED 410.

The absorption band edge of ZnO NPs lies within the 370–390 nm range, with their visible-spectrum absorption attributed to defect states that introduce levels within the band gap. For irradiation, we selected LEDs at 375 nm (near-UV, band edge) to facilitate absorption, and 410 nm (blue visible region) to excite defect sublevels in the ZnO structure. Figure 5 presents data on bacterial inactivation under irradiation, both with and without ZnO nanoparticles. In all cases, exposure to 0.05 g/L ZnO NPs and LED light, regardless of power, resulted in a reduction in *S. aureus* concentration.

Exposure to near-UV LED375 irradiation at 0.17 W reduced the concentration of *S. aureus* bacteria by two orders of magnitude, from 10^6^ to 10^4^ CFU/mL. Increasing the irradiation power to 0.38 W resulted in a further reduction to 10^3^ CFU/mL (Figure 5a). Similarly, irradiation with blue-visible LED410 at 0.22 W decreased bacterial concentration by approximately two orders of magnitude, comparable to the effect of LED375. Increasing the LED410 power to 0.36 W led to an additional 5–6-fold reduction after 2 h of exposure (Figure 5b).

When ZnO NPs were combined with near-UV LED375 irradiation, 100% bacterial eradication occurred within 2 h at both optical powers tested (0.17 and 0.38 W). Similarly, exposure to blue-visible LED410 radiation at 0.22 W for 2 h reduced *S. aureus* concentration by three orders of magnitude to 10^3^ CFU/mL. Increasing the blue-visible irradiation power to 0.36 W in the presence of ZnO NPs resulted in complete bacterial elimination.

#### 3.2.4. Effect of Ag Dopant on Antibacterial Activity of ZnO NPs

Modification of ZnO NPs with silver particles was found to enhance absorption in the visible spectrum (Figure 3a), suggesting increased activity under visible-light LED410 irradiation. Additionally, in ref. [50], we demonstrated that excitation of ZnO-1Ag composite NPs within the silver SPR band enhanced their photocatalytic activity. While silver NPs are widely used for antibacterial applications, high concentrations can lead to adverse effects [70]. That is why, in this study, we incorporated a small amount of silver (1%) to modify laser-produced ZnO NPs.

**Figure 6:**
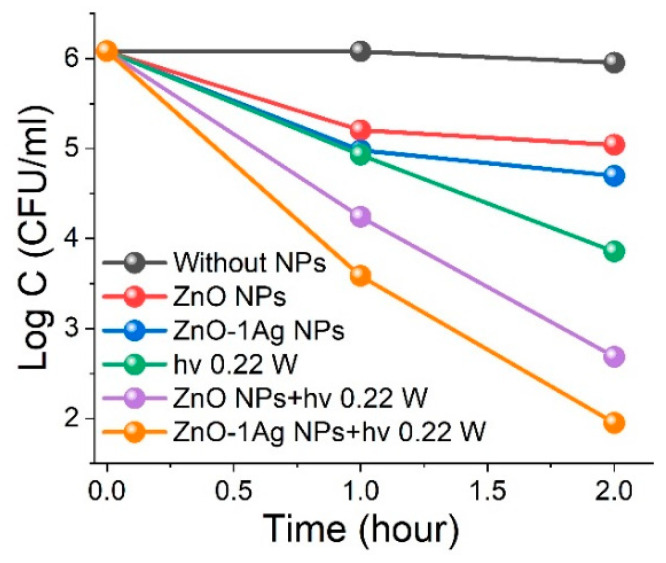
Effect of Ag addition on antibacterial activity of ZnO without and with LED410 irradiation.

Without irradiation, 0.05 g/L ZnO NPs reduced *S. aureus* concentration by approximately one order of magnitude, from 10^6^ to 10^5^ CFU/mL. The incorporation of ZnO-1Ag NPs at the same concentration further increased antibacterial efficacy, reducing bacterial counts by an additional 2–3 times (Figure 6). Exposure to LED410 radiation at 0.22 W alone decreased bacterial concentration from 10^6^ to 10^4^ CFU/mL, while the combined effect of irradiation and 0.05 g/L ZnO NPs reduced the count further to 6 × 10^2^ CFU/mL (see Figure 5b and Figure 6). The combination of ZnO-1Ag NPs, modified with 1% silver, and low-power LED410 irradiation at 0.22 W significantly enhanced antibacterial activity, resulting in a bacterial concentration of only ~6 × 10^1^ CFU/mL after 2 h (see Figure 6).

**Figure 7:**
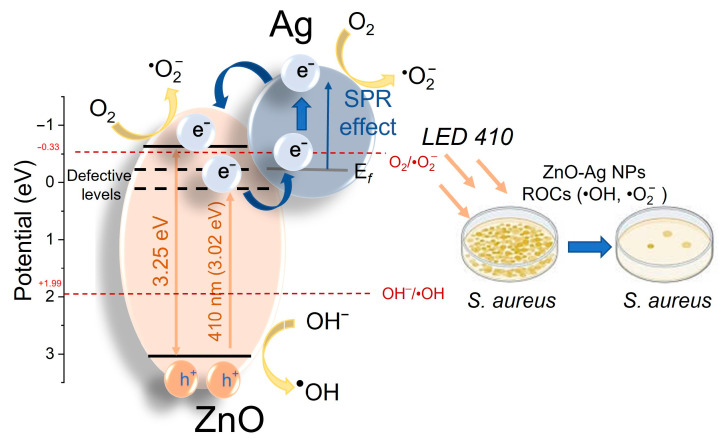
Scheme of charge carrier transfer in NPs of ZnO and ZnO-1Ag upon LED 410 irradiation.

The observed enhancement in the antibacterial activity of ZnO-1Ag NPs is attributed to the synergistic effects of ZnO structural defects and the influence of Ag SPR. The mechanisms underlying the antibacterial activity of semiconductor ZnO NPs and ZnO-1Ag composite particles under photoexcitation are illustrated in Figure 7. The photocatalytic antibacterial effect of wide-bandgap semiconductor NPs is known to be driven by the generation of reactive oxygen species (ROS) [37]. Pure ZnO is known to readily generate hydroxyl radicals (•OH) with a high oxidative capacity (1.4 V, for neutral medium), whereas electron paramagnetic resonance (EPR) studies showed that the presence of sulfur could suppress (quench) this specific pathway [71]. Instead, the composite may preferentially promote the formation of superoxide radicals (•O_2_^−^, with an oxidative potential of 0.93 V, for neutral medium) due to more efficient electron transfer at the Ag-ZnO interface [54,71,72]. The theoretical positions of the valence and conduction bands of ZnO were calculated using a previously reported formula [73,74] and compared with the formation potentials of •OH and •O_2_^−^ ROS [11]. 

Under LED410 irradiation, the photon energy is insufficient for an electron to undergo an inter-band transition in ZnO NPs (E_g_ = 3.25 eV). Consequently, photogenerated electrons are captured by defect states in ZnO (more specifically, interstitial oxygen and zinc atoms in both ground and ionic states) located near the conduction band. These defect-trapped electrons contribute to ROS generation and can also transfer to nanoparticles or Ag clusters, thus leading to the formation of a Schottky barrier, characteristic of noble metals with high work functions [36,43]. Additionally, LED410 irradiation excites electrons within silver particles/clusters on the ZnO surface, which further participate in ROS generation or migrate to the ZnO conduction band. This leads to a significant increase in ROS production, which causes oxidative stress, damages cellular proteins, lipids, and DNA, eventually resulting in bacterial death. Thus, Ag incorporation enhances absorption and charge separation, improving ROS generation efficiency under photoexcitation and thereby increasing *S. aureus* bacterial inactivation [41].

It is worth noting that bacterial death can also occur due to the action of Ag^+^ ions present in solution [75,76]. However, silver leaching is believed to be significantly limited in our experiments, which were conducted in phosphate-salt buffer (PBS). The high concentration of chloride ions (~137 mM) in PBS favors the deposition of insoluble AgCl on the NP surface. Moreover, our method of co-precipitation of Ag with ZnO, followed by calcination at 400 °C, promotes strong silver adhesion and formation of a stable heterojunction at the interface. This is also believed to play an important role in the immobilization of silver and the limitation of its leaching into solution. 

Table 2 presents a comparison of the antibacterial activity of various ZnO and ZnO-Ag materials upon photoactivation, based on publications that employed similar experimental conditions for bacterial concentration, growth, and analysis. Notably, studies utilizing disk-diffusion and droplet methods were excluded from consideration [41,51,52,53,77,78,79,80]. The data demonstrate that the ZnO and composite ZnO-1Ag NPs synthesized in this work exhibited excellent antibacterial efficacy, even at low nanoparticle concentrations, when combined with low-power UV-A and blue visible radiation sources.

## 4. Conclusions

In this study, we synthesized composite ZnO-Ag nanoparticles using the pulsed laser ablation method in water and evaluated their antibacterial performance in comparison to silver-free ZnO nanoparticles prepared under similar conditions.

The study confirmed that highly defective ZnO nanoparticles effectively inhibited *S. aureus* bacteria even at low concentrations (0.05 g/L). The abundance of defect states within the band gap increased absorption in the visible spectrum. Photoluminescence analysis identified the defect states, revealing charge carrier transfer between interstitial zinc and excess oxygen atoms (both ground and ionic states) and the valence and conduction bands of ZnO nanoparticles. Furthermore, irradiation with low-power (0.17–0.38 W) near-UV (375 nm) and blue visible (410 nm) light enhanced the antibacterial activity of semiconductor ZnO particles by promoting additional photocatalytic generation of reactive oxygen species in the medium.

The introduction of low concentrations of silver into ZnO-Ag composite nanomaterials led to increased absorption in the visible spectrum, attributed to the surface plasmon resonance of Ag. Additionally, silver incorporation improved the separation of photogenerated charges within the composite particles, enhancing reactive oxygen species generation and further boosting antibacterial activity. Consequently, the combined use of ZnO composite particles with 1% silver content and irradiation with low-intensity, safe LED light in the near-UV and blue visible range significantly improves bacterial inactivation efficiency while reducing the required concentration of antibacterial particles.

## Figures and Tables

**Table 1:** Characteristics of PLA-generated NPs.

Sample	Phase Composition		Lattice Parameters, Å	CSR, nm	Ag Content, wt.% *	BET Surface Area (m^2^/g)	Band Gap (eV)
	Phase	Content, %					
ZnO	ZnO	100	*a* = *b* = 3.2489 *c* = 5.2049	43	–	21	3.25
ZnO-1Ag	ZnO	99	*a* = *b* = 3.2484 *c* =5.2009	37	0.98	26	3.24
	Ag	1					

* according to XRF data.

**Table 2:** Comparison of light-enhanced antibacterial activity of ZnO and Ag/ZnO materials.

Antibacterial Material (Synthesis Method) C (g/L)	Bacterium C (CFU/mL)	Experimental Environment/ Light Source Parameters/ Irradiation Time	Residual Survival	Ref.
ZnO (sol-gel) 10	*S. aureus* 10^6^	LB culture medium/ Vis LED 600 nm/ 3 h	ZnO + *hv*—3.57%	[41]
	*E. coli* 10^6^		ZnO + *hv*—4.28%	
Commercial (Riedel-de Haën) ZnO 2	*E. coli* 10^7^	LB culture medium/ UV lamp 365 nm, 20 W/m^2^/ 40 min	only *hv*~10^6^ CFU/mL ZnO + *hv*~1 CFU/mL	[77]
	*L. helveticus* 10^6^		only *hv*~5 × 10^6^ CFU/mL ZnO+ *hv* 2 × 10^2^ CFU/mL	
ZnO of different shapes (co-precipitation) 2	*P. aeruginosa* 10^7^	PBS solution/ Sunlight/ 45 min	only hv~10^6^ CFU/mL ZnO (flower-shaped) + *hv*—CR ZnO (other shapes) + *hv*~3—50 CFU/mL	[78]
ZnO NPs of different shapes (ice-cube mediated synthesis) 0.5	*E. coli* 10^4^	PBS solution/ Sunlight (India10.07 °N-78.80 °E)/ 30–75 min	ZnO + *hv*—CR	[51]
Ag/ZnO (solvothermal) ~0.01	*E. coli* ~10^5^	PBS solution/ UV light/ 2 h	Ag/ZnO + *hv*—CR	[79]
	*S. aureus* ~10^5^		Ag/ZnO + *hv*—CR	
BIN ZnO 0.006–0.01	*E. coli* ~10^5^	Nutrient broth/LED (7W)/2 h	BIN ZnO + *hv*—CR	[80]
	*S. aureus* ~10^5^			
			BIN ZnO + *hv*—CR	
ZnO and ZnO/Ag NPs in matrix	*E. coli* ~10^5^	Nutrient medium/low-intensity UVA illumination/2 h	ZnO~10^2^ CFU/cm^2^ and ZnO/Ag~10^2^ CFU/cm^2^	[52]
	*S. aureus* ~10^5^			
			ZnO~10–10^2^ CFU/cm^2^ and ZnO/Ag~10^4^ CFU/cm^2^	
ZnO (flower- shaped) and Ag/ZnO (co-precipitation) 1	*E. coli* ~10^7^	Sterile saline water/Vis light source/180 min	ZnO–<50% CFU Ag/ZnO–CR	[53]
ZnO, ZnO-1Ag (PLA) 0.05	*S. aureus* 10^6^	PBS solution/ LED 375 nm, 0.17 W, 0.38 W and LED 410 nm, 0.22, 0.36 W/ 2 h	only *hv* 375 nm 0.38 W~103 CFU/mL only *hv* 410 nm 0.36 W~3 × 10^3^ CFU/mL ZnO + *hv* 375 nm 0.17 W—CR ZnO + *hv* 410 nm 0.36 W—CR ZnO + *hv* 410 nm 0.22 W~2.8 × 10^2^ CFU/mL ZnO-1Ag + *hv* 410 nm 0.22 W—6 × 10^1^ CFU/mL	The Work

CR: Complete Removal.

## Data Availability

The original contributions presented in this study are included in the article/Supplementary Materials. Further inquiries can be directed to the corresponding author.

## Supplementary Materials

The following supporting information can be downloaded at: https://www.mdpi.com/article/10.3390/ma18133088/s1. The Supporting Information contains Figure S1. X-ray diffraction of ZnO and ZnO-1Ag samples and Figure S2. Evolution of bacteria *S. aureus* in sodium phosphate buffer (PBS).
