# Supplementary material for: Laser-Prepared ZnO-Ag Nanoparticles with High Light-Enhanced Antibacterial Activity
Source: Materials (Basel). 2025 Jun 29;18(13):3088. doi: 10.3390/ma18133088 (PMC12251156; doi:10.3390/ma18133088)
Supplement: Supplementary file 1 [file materials-18-03088-s001.zip › materials-3671683-supplementary.pdf]

Supplementary Information for:

Laser-Prepared ZnO-Ag Nanoparticles with High Light-Enhanced Antibacterial Activity

Anastasia V. Volokitina [1], Elena D. Fakhrutdinova [1], Daria A. Goncharova [1], Sergei A. Kulinich [2,*] and Valery A. Svetlichnyi [1,*]

[1] Laboratory of Advanced Materials and Technology, Tomsk State University, 634050 Tomsk, Russia

[2] Research Institute of Science & Technology, Tokai University, Hiratsuka, Kanagawa 259-1292, Japan

*skulinich@tokai.ac.jp; v_svetlichnyi@bk.ru

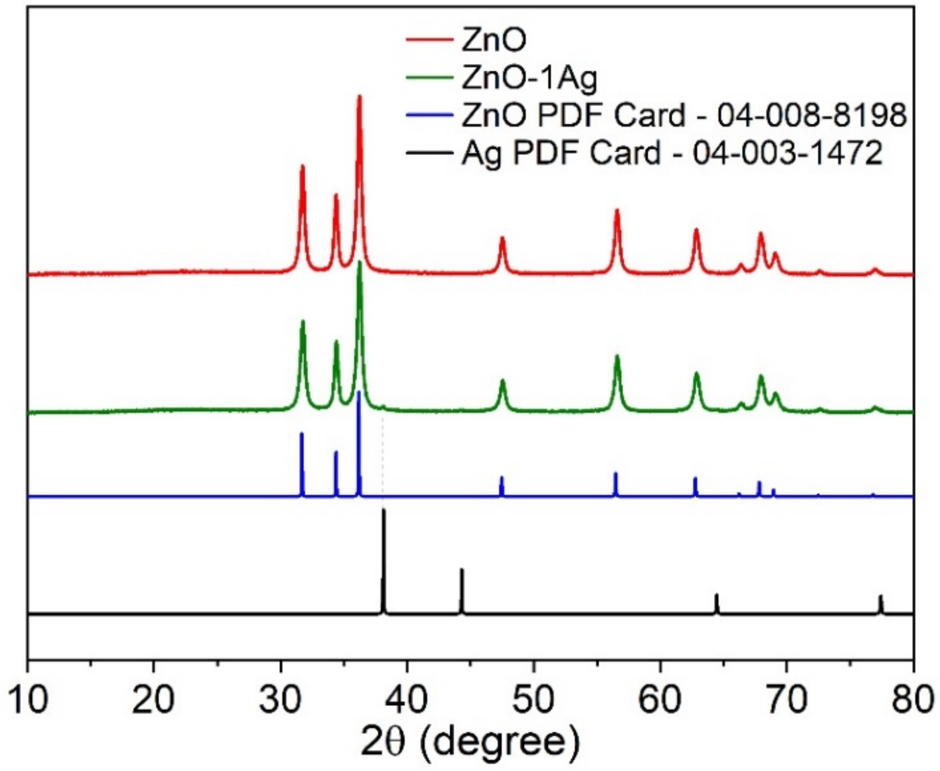

Figure S1. XRD patterns of samples ZnO and ZnO-1Ag.

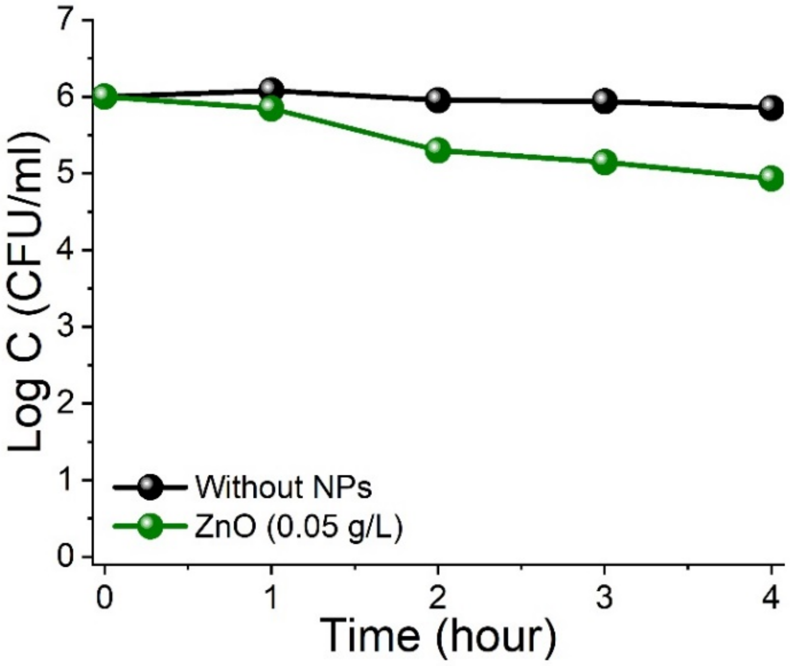

Figure S2. Evolution of bacteria *S. aureus* in sodium phosphate buffer (PBS).
